# In vitro models of the choroid plexus and the blood-cerebrospinal fluid barrier: advances, applications, and perspectives

**DOI:** 10.1007/s13577-024-01115-5

**Published:** 2024-08-05

**Authors:** Christian Schwerk, Horst Schroten

**Affiliations:** 1 Pediatric Infectious Diseases, Department of Pediatrics, Medical Faculty Mannheim, Heidelberg University, 68167 Mannheim, Germany; 2 European Center for Angioscience, Medical Faculty Mannheim, Heidelberg University, 68167 Mannheim, Germany

**Keywords:** Blood–cerebrospinal fluid barrier, Choroid plexus, Microfluidic, Organoid, Organ-on-a-chip

## Abstract

The choroid plexus (CP), a highly vascularized endothelial–epithelial convolute, is placed in the ventricular system of the brain and produces a large part of the cerebrospinal fluid (CSF). Additionally, the CP is the location of a blood–CSF barrier (BCSFB) that separates the CSF from the blood stream in the CP endothelium. In vitro models of the CP and the BCSFB are of high importance to investigate the biological functions of the CP and the BCSFB. Since the CP is involved in several serious diseases, these in vitro models promise help in researching the processes contributing to the diseases and during the development of treatment options. In this review, we provide an overview on the available models and the advances that have been made toward more sophisticated and “in vivo near” systems as organoids and microfluidic lab-on-a-chip approaches. We go into the applications and research objectives for which the various modeling systems can be used and discuss the possible future prospects and perspectives.

## Introduction

### The choroid plexus and the inner blood–cerebrospinal fluid barrier

The central nervous system (CNS) is a highly sensitive structure that requires a specific milieu for proper function and needs to be protected against damage by influences from outside of the CNS. One mechanism to shelter the CNS is the surrounding of the brain and the spinal cord by a liquid layer of cerebrospinal fluid (CSF). A large part of the CSF is produced by the choroid plexus (CP), a highly vascularized organ that is located in the ventricles, which constitute CSF-filled excavations in the brain. The structure of the CP includes an outer epithelial layer formed by cells that exhibit extensive microvilli, strongly enhancing the cellular surface bordering the CSF in the ventricles, and endothelial cells that are responsible for an extensive vascularization. Further cells present in the CP are immune cells as dendritic cells, NK cells, lymphocytes as T cells, and macrophages, which (together with the vasculature) are embedded in the CP stroma. Macrophages termed Kolmer epiplexus cells are also located apically at the CP epithelium [1–3].

Due to its location in the ventricles, the CP presents a direct interface between the CSF and the blood, and therefore between the CNS and the remainder of the organism. To avoid that substances and also pathogens can unhindered cross the CP to enter the CNS, a barrier has to be present at the CP. For this purpose, the epithelial cells of the CP are connected to each other by tight junctions (TJs) that form tight strands and seal the epithelial layer, thereby generating the so-called inner blood–CSF barrier (BCSFB) [4]. Although the vasculature at the CP consists of fenestrated endothelial cells that have long been thought not to contribute to barrier function, more recent data indicate that the CP endothelium can modulate the barrier at the CP [5, 6].

### Multiple functions of the CP

The CP fulfills several fundamental roles in the organism that are central to brain function, as exemplary the production and secretion of the lion’s share of the CSF. Besides providing a “physical buffer” for the brain, the CSF is required to guarantee the maintenance of brain homeostasis and of the intracranial pressure. Correct amounts of CSF with a defined composition are generated due to the presence of specific transporters and the production of proteins as transthyretin by the CP [7–9]. These transporter systems also contribute to the barrier function of the CP epithelium by selected and directed transport of large amounts of substances. The presence of the transporters helps to transport toxic substances out of the brain, but importantly also presents a major obstacle for the brain transport of pharmaceutical molecules during treatment of CNS diseases [10, 11]. A low pinocytotic activity and the presence of dense TJ strands complement the mechanisms to mediate the barrier function of CP epithelial cells [12, 13].

The barrier function is essential to protect the brain from inflammatory molecules, toxins, and several kinds of pathogens that can be present in the bloodstream [14, 15]. Another important function of the CP barrier function is the regulation of the entry of immune cells into the CNS [3, 16, 17]. This regulation is integral in modulating brain immunity under healthy conditions, but immune cell transmigration into the CNS also plays a central role during several diseases of the CNS [18, 19].

### Involvement of the CP in diseases

In addition to its multiple functions under healthy condition, the CP is also well known to play a role during a multitude of diseases [2, 14, 20–22]. There is evidence that the CP and the BCSFB respond to traumatic brain injury, and data suggest that CSF hypersecretion by the CP may contribute to post-hemorrhagic or post-infectious hydrocephalus [20, 21, 23]. Involvement of the CP was also shown for neurodegenerative disorders as Alzheimer’s disease and Parkinson’s disease [20–22].

Much attention has been paid to the roles of the CP and the BCSFB during autoimmune disorders, in particular concerning multiple sclerosis, where the CP can serve as entry gate for immune cells into the CNS [18, 20, 21]. The CP is also involved in infectious diseases of the CNS, since several types of pathogenic organisms can enter the brain across the BCSFB. These pathogens include viruses, bacteria, fungi, and parasites that, following CNS invasion, cause an inflammatory response culminating in meningitis, encephalitis, and meningoencephalitis. Host immune cells that subsequently to infection enter the brain, again including across the BCSFB as entry gate, contribute substantially to the inflammatory reactions and damage [15, 17, 23, 24].

The CP can also develop tumors that range from papillomas that cytologically and architecturally closely resemble the normal CP to carcinomas displaying a morphology without resemblance to the healthy CP. As for immune cells, the CP can also serve as a portal for tumor cells, including neuroblastoma and leukemia cells, to get access to the CNS with subsequent formation of metastasis [20, 25, 26]. Finally, the CP–CSF interface has also been implicated in neuropsychiatric diseases such as schizophrenia and autism [27].

**Fig. 1:**
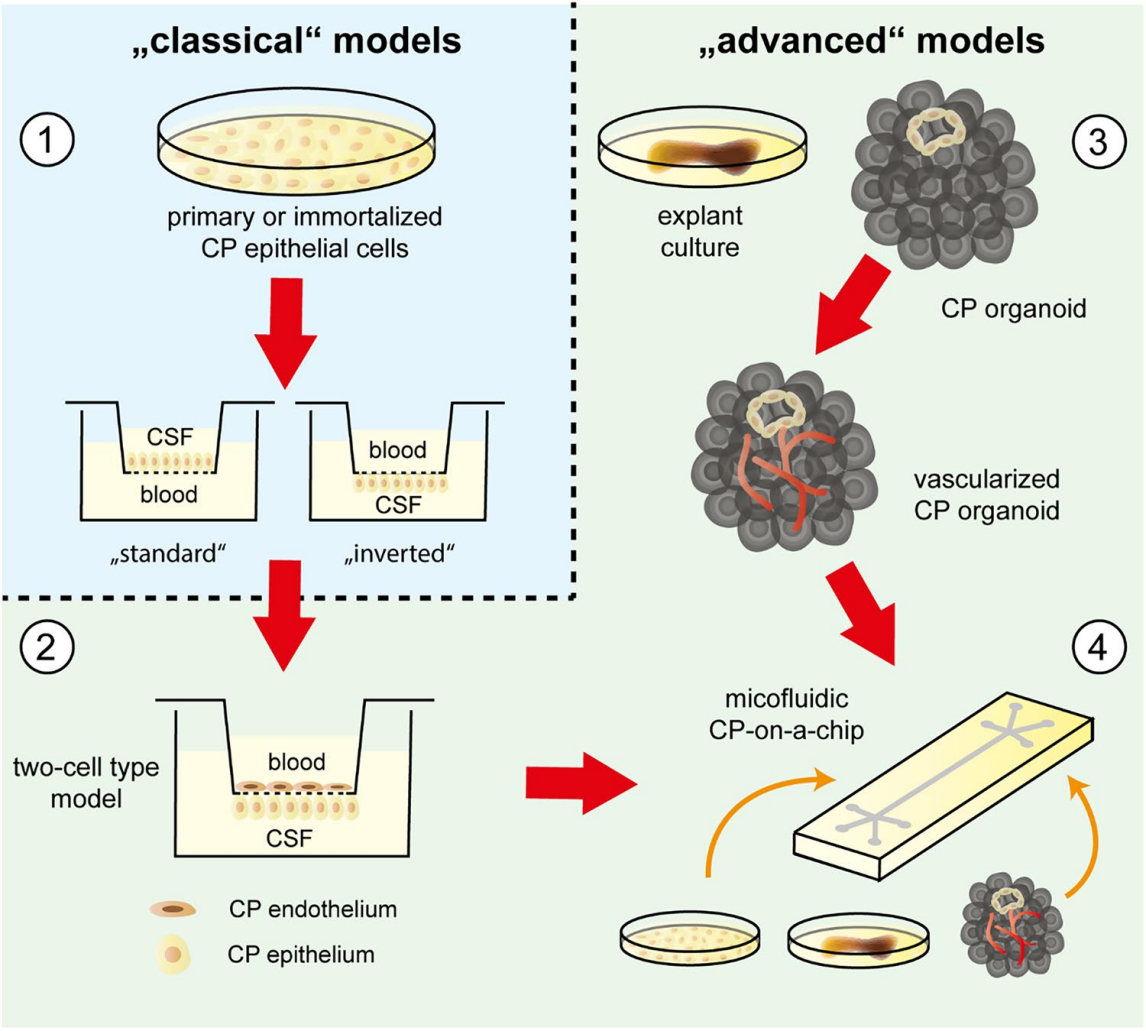
Overview of available CP and BCSFB in vitro models. **1** “Classical” models of the BCSFB are mostly based on primary or immortalized CP epithelial cells that can be grown on cell culture filter insert supports for generation of a barrier, separating a “CSF” compartment from a “blood” compartment. The orientation of the two compartments in the model system depends on whether the cells are cultivated on the upper side (“standard” model) or the lower side (“inverted” model) of the filter membrane. **2** A first step toward an “advanced” model is the integration of further CP cell types, as the endothelium constituting the vasculature in the CP. A two-cell type model of the CP can be generated by growing CP endothelial cells on the upper side and CP epithelial cells on the lower side of the membrane of cell culture filter inserts. **3** CP explant cultures and organoids with CP-like features (CP organoids) present a detailed 3D structure resembling the CP in vivo. Strategies have been developed to vascularize organoids to overcome the disadvantage of a lacking vasculature. **4** Microfluidic organ-on-a-chip model systems further consider fluid movements that impact on cellular structures. Incorporation of CP cells, explants, and organoids into organ-on-a-chip models promises the generation of highly advanced CP and BCSFB in vitro models

Involvement of the CP in the described multitude of diseases, additionally to the functions under healthy conditions, is a major reason for the necessity of appropriate in vitro models of the BCSFB for use in basic research, drug testing and drug development, and permeability studies. An overview of the available in vitro models of the CP and the BCSFB is given in Fig. 1 and will be summarized in detail in the following chapter.

## In vitro models of the choroid plexus and the blood–cerebrospinal fluid barrier

### “Classical” models

When generating models of the CP and the BCSFB, researchers have often focused on CP epithelial cells, which are to a large part responsible for major functions of the CP including barrier function (based on TJs and transporter systems) and the production of CSF. Primary CP epithelial cells have been prepared from several species as rodents, pigs, and non-human primates, and tend to retain these major functions to a large extent, e.g., by providing a sufficient barrier function for in vitro studies of the BCSFB when grown on cell culture filter inserts [28–32]. Furthermore, CP epithelial cells of human origin (HCPEpiC) are commercially available. Since primary cells are hard to obtain in large numbers, can only be cultured for a limited range of passages, and are often difficult to manipulate genetically, they are only sub-optimally suited to achieving certain research objectives. To overcome these obstacles, immortalized cell lines have been generated, which, on the other hand, often do not faithfully recapitulate major properties of the CP epithelium as barrier function or CSF production [33–38]. CP epithelial cell lines with strong barriers that are derived from pig and human have been described [38, 39].

The CP epithelial cells can be grown on membrane supports provided by cell culture filter inserts to create a system consisting of two compartments, a “CSF” compartment and a “blood” compartment. The experimenter has the choice to culture the cells on the upper side (“standard” model) or the lower side (“inverted” model) of the membrane, dependent on the desired orientation of the two compartments [40, 41]. These “classical” models of the BCSFB have been used with success for studies of drug transport and the pathology of several diseases of the CNS [10, 15, 30]. Still, these models only partly reflect the in vivo CP, since they only consist of epithelial cells and the other cell types contained in the CP are missing. Also, the CP has a specific morphology that is only partially mimicked by growth of CP epithelial cells on cell culture filter inserts.

### “Advanced” models

#### Integrating additional cell types

An obvious step to advance in vitro models is to integrate additional cell types. Concerning the BCSFB, the endothelial cells of the CP are of major interest, since the CP is highly vascularized and recent research has indicated a role of the CP endothelium during modulation of barrier function [5, 6]. It is known that endothelial cells of distinct organs display specific properties that distinguish them from other endothelia [42]. The endothelial cells of the human CP form a fenestrated endothelium characterized by the expression of the plasmalemma vesicle-associated protein (PLVAP) and the presence of caveolae and fenestrae [43]. The recent generation of immortalized choroid plexus endothelial cells (iHCPEnC), which retain major characteristics of the CP endothelium in vivo, enabled the set-up of a two-cell type model of the BCSFB that consists of CP epithelial and endothelial cells grown on opposite sides of cell culture filter inserts. This model displays an enhanced barrier function compared to a model based on epithelial cells alone [6], and holds significant promise for advanced studies of the BCFSB in vitro, especially concerning the endothelial–epithelial interplay at the CP under healthy and pathological conditions.

#### Explants, stem cells, and organoids

The model systems described so far consist of isolated primary cells or generated cells lines representing components of the CP. These models certainly have the advantage that specific research questions can be addressed in rather defined experimental settings consisting of selected cell types. Still, despite an increased complexity obtained by combining different cell types, several disadvantages further exist as the lack of a shear stress caused by flow and the absence of a detailed 3D structure resembling the CP in vivo.

To overcome some of these disadvantages, more advanced 3D-culture models have been developed. Explant cultures are based on tissue dissected from CP material, e.g., human tissue taken postmortem or during surgery, or tissue taken from different animal models [44]. Tissues representing the CP obtained from rats and mice, from guinea pig, and from shark have been used to study the location of several transporters and receptors, transport processes, or the migration of immune cells, respectively [45–50]. A disadvantage of these explant cultures is, however, that they are mostly derived from non-primates as rodents.

Limitations of the BCSFB in vitro models based on primary cells, cell lines, or explant cultures can be overcome with the help of stem cells that are induced to represent CP tissue. Bone morphogenetic protein 4 (BMP4) was sufficient to derive CP epithelium from mouse and human neuroepithelial stem cells, and employing both BMP4 and Wnt signaling strongly induced choroid plexus-like tissues from human embryonic stem cells in 3D culture [51, 52]. When taken into culture, the induced stem cells can be grown further into cellular assemblies that recapitulate the structure of organs. These so-called organoids are defined as 3D structures that by self-assembly and differentiation are able to mimic at least some functions of defined organs [53]. 3D organoids derived from human pluripotent stem cells were established that presented discrete brain regions including the CP [54, 55]. Treatment with BMP4 in combination with Wnt activation after the organoids were embedded in Matrigel lead to structures enriched in cuboidal epithelium representing polarized CP epithelial cells. Notably, these CP organoids presented a robust barrier function and developed compartments filled with a CSF-like fluid [56]. Recently, brain organoids consisting of a core of functional cortical neurons that are surrounded by an epithelium presenting CP-like features have been generated using an induced pluripotent stem cell line derived from a patient with Down syndrome and its isogenic euploid counterpart, respectively [57].

One disadvantage of the CP organoids described above is that they lack a vasculature. This restriction leads to cellular stress and cell death due to hypoxia and lack of nutrient, and limits the size of the organoids. Also, endothelial cell signaling is missing, which is required for correct organoid development [58]. Several approaches to vascularize brain organoids have been published. These include the transplantation of organoids into the brains of immunodeficient rodents, the addition of vasculature-deriving cells, co-culture of human umbilical vein endothelial cells (HUVECs) with induced pluripotent or embryonic stem cells for the production of organoid precursors that, following neural induction, display a vascular system, and co-culture of brain organoids with blood vessel organoids [58, 59]. Furthermore, the heterogeneity of different types of brain endothelia should be considered. To generate an organoid model with an “in vivo near” vascularized CP, the use of CP-specific endothelial cells is advisable—conceivably without or together with other types of brain endothelia as the microvascular endothelial cells of the BBB. It can be anticipated that with ongoing research progress, highly advanced CP organoid models will be available.

#### Microfluidic “choroid plexus-on-a-chip” models

To faithfully mimic the physiology of organs, it is necessary to consider the fluid movements that impact on the respective cellular structure, as the flow of blood through endothelial vessels. So-called “organ-on-a-chip” microfluidic devices have been invented, in which living cells can be cultured under fluid flow conditions that recapitulate the in vivo conditions [60].

A human microfluidic BCSFB model has recently been described that consists of commercially available CP epithelial and brain microvascular endothelial cells, which can be exposed to medium mimicking the dynamic flow of blood and CFS [61]. In this model, the architecture of the BCSFB could be reproduced, exemplified by the presence of TJs and the formation of a physiologically relevant permeability for macromolecules. When this model was exposed to inflammatory stimuli as tumor necrosis factor (TNF)-α, neuropathological consequences as the regulation of key innate immunity response genes and barrier damage were observed. Still, the authors did not achieve a co-culture with immune cells, which would be important, e.g., for the study of immune cell transmigration across the BCSFB [61]. Also, the use of CP-specific endothelial cells instead of brain microvascular endothelial cells should be better suited to correctly model the CP and the BCSFB. In another approach, Lim and coworkers generated a microfluidic chip that was used to reconstitute the CP with commercially available human brain microvascular endothelial cells, pericytes, and CP epithelial cells on an engineered extracellular matrix and under dynamic conditions mimicking the CSF flow in vivo [62]. In this system, the authors could show physiologically relevant drug responses by breast cancer cells that were added to the model. Furthermore, immune responses in the CP were recapitulated by applying macrophages, the most common immune cells in the CP, to the system.

Generally, BCSFB-on-chip models and other model systems as organoids represent distinct approaches and can supplement each other. In this regard, the application of microfluidics is not limited to BCSFB models based on single or multiple cell types, but can just as well employed to improve the quality and usefulness of organoid models that have been subjected to vascularization [63].

## Applications of the in vitro models

The in vivo properties of the CP should be mirrored by in vitro models as exactly as possible to enable their use as research tools on CP functions. Models that faithfully reproduce the major tasks of the CP will be very helpful in elucidating the “biology” of the CP. Here, interesting aspects concern the interplay between different cell types present in the CP, e.g., during development, maturation, and aging [64]. Noteworthy, suitable models that produce CSF-like fluids [29, 56] can be used to study the functions of a secretory epithelium. Since in vitro organoids and CSF-like fluid can mature to a state strongly resembling postnatal stages or adulthood, they might allow the identification and investigation of disease-related biomarkers [56].

The involvement of the CP in diseases has been already addressed in this review. Suitable models of the CP will help to investigate the development of these diseases, including CP papillomas and carcinomas [65]. In vitro models of the CP and the BCSFB have also been intensively used to research the pathogenetic processes during infectious diseases of the CNS caused by viruses, bacteria, and parasites [41, 66–69]. In this regard, experiments taking advantage of organoids have contributed to elucidate the involvement of the CP by viral diseases that came recently into focus as Zika virus and SARS-CoV-2 [68, 70, 71]. Interestingly, the application of organoids with a functional CP-like epithelium generated from an induced pluripotent stem cell line derived from a down syndrome patient has shown that neurotropism of SARS-CoV-2 is enhanced by CP defects in Down syndrome brain organoids [57].

The CP is also a regulatory gate for entry of immune cells into the CNS. During the course of diseases as multiple sclerosis and as response to infections of the brain, host immune cells enter the CNS and cause substantial damage. Mechanisms of immune cell traversal across the CP, e.g., interactions between immune cell and barrier cell surface proteins and migration pathways (paracellular and transcellular), have been investigated and deciphered in cell culture insert and explant systems recapitulating the CP and the BCSFB [50, 72]. The same models can be used to study the migration of cancer cells into the CNS across the BCSFB [26].

As one of the barriers separating the CNS from the blood, the BCSFB at the CP presents a major obstacle for the delivery of pharmaceutical substances into the brain for the treatment of diseases. In vitro models of the CP presenting an appropriate BCSFB, including cell culture filter-based systems as well as organoids, can be used to develop and evaluate brain accessible (neuro)pharmaceuticals for disease treatment and prevention, which should help to reduce the number of drug candidates that fail during clinical testing [10]. In this regard, expression of transporter proteins has been demonstrated in CP and BCSFB in vitro models [50, 73, 74]. Recently, the extracellular vesicle biogenesis of CP organoids was investigated and also supported CP organoids as a model system for screening of drugs and development of drug delivery systems for treatment of neurological disorders [75].

## Perspectives

Significant progress has been made during the development of in vitro CP and BCSFB model systems, and the quantity of possible and established applications is steadily increasing. Still, there are further tasks that are worth to pursue to increase the quality of the models and to widen their spectrum of research capabilities. We have already discussed the necessity, options, and attempts to include vasculature into the existing CP models. Another important component that deserves consideration is the integration of immune cells, especially since the CP is rated as an important gateway for immune cells into the CNS during health and disease [3, 16, 17].

One interesting step toward more complete in vitro systems would be to combine CP models with further structures, specifically CNS components, but also others. In this direction, a neurovascular unit with a complex 3D structure has been constructed by organ-on-a-chip technology that considers a “CSF”-side, but does not contain CP cells [76]. Furthermore, Alcendor and colleagues have suggested an organ-on-a-chip model that contains, besides a BBB and a brain–CSF barrier, also a BCSFB [77]. As pointed out by Ye, the combination of vascularized CP organoids with vascularized brain organoids presenting further CNS structures could have the added advantage to generate a more complete vasculature [58].

Further promising perspectives for the use of CP and BCSFB models will concern the evaluation of molecular mechanism of diseases and possible treatment. Besides the detection of potential biomarkers, advanced model systems as organoids can be employed for use in personalized medicine. The use of stem cells from patients suffering from neurological disorders as autism spectrum disorders, Parkinson’s disease, or Alzheimer’s disease allows the generation of organoids for individual disease modeling and testing of therapies in a personalized manner [78]. It is conceivable that this approach can be successfully adapted for CP organoids.

Finally, it should be emphasized that organoid models are major candidates in replacing laboratory animals. In this regard, the CP organoid developed by Pellegrini and colleagues [56] has won the 2020 3Rs Prize, awarded by the NC3Rs and co-funded by GSK (https://www.nc3rs.org.uk/news/cerebral-organoid-model-wins-3rs-prize). It is to be expected that future advanced CP and BCSFB models will further contribute to the important task of reducing the amount of laboratory animals.

## Conclusions

Several in vitro model systems of the CP and the BCSFB have been developed that can be employed to investigate biological CP functions in health and disease. With the improvement of these models toward more complex and “in vivo-like” conditions, their research options and applications will increase. It is promising that future advanced systems, e.g., organoid models and lab-on-a-chip approaches, can be employed for personalized medicine and will help to reduce animal experimentation.

## References

[1] Kratzer I, Ek J, Stolp H. The molecular anatomy and functions of the choroid plexus in healthy and diseased brain. Biochim Biophys Acta Biomembr. 2020;1862(11): 183430. 32750317 10.1016/j.bbamem.2020.183430

[2] Ghersi-Egea JF, Strazielle N, Catala M, Silva-Vargas V, Doetsch F, Engelhardt B. Molecular anatomy and functions of the choroidal blood-cerebrospinal fluid barrier in health and disease. Acta Neuropathol. 2018;135(3):337–61. 29368213 10.1007/s00401-018-1807-1

[3] Castellani G, Croese T, Peralta Ramos JM, Schwartz M. Transforming the understanding of brain immunity. Science (New York, NY). 2023;380(6640):eabo7649. 10.1126/science.abo7649 37023203

[4] Saunders NR, Dziegielewska KM, Mollgard K, Habgood MD. Physiology and molecular biology of barrier mechanisms in the fetal and neonatal brain. J Physiol. 2018;596(23):5723–56. 29774535 10.1113/JP275376 PMC6265560

[5] Carloni S, Bertocchi A, Mancinelli S, Bellini M, Erreni M, Borreca A, et al. Identification of a choroid plexus vascular barrier closing during intestinal inflammation. Science (New York, NY). 2021;374(6566):439–48. 10.1126/science.abc6108 34672740

[6] Muranyi W, Schwerk C, Herold R, Stump-Guthier C, Lampe M, Fallier-Becker P, et al. Immortalized human choroid plexus endothelial cells enable an advanced endothelial-epithelial two-cell type in vitro model of the choroid plexus. iScience. 2022;25(6):104383. 35633941 10.1016/j.isci.2022.104383 PMC9133638

[7] Spector R, Robert Snodgrass S, Johanson CE. A balanced view of the cerebrospinal fluid composition and functions: focus on adult humans. Exp Neurol. 2015;273:57–68. 26247808 10.1016/j.expneurol.2015.07.027

[8] Praetorius J, Damkier HH. Transport across the choroid plexus epithelium. Am J Physiol Cell Physiol. 2017;312(6):C673–86. 28330845 10.1152/ajpcell.00041.2017

[9] MacAulay N, Keep RF, Zeuthen T. Cerebrospinal fluid production by the choroid plexus: a century of barrier research revisited. Fluids Barriers CNS. 2022;19(1):26. 35317823 10.1186/s12987-022-00323-1 PMC8941821

[10] Dabbagh F, Schroten H, Schwerk C. In vitro models of the blood-cerebrospinal fluid barrier and their applications in the development and research of (neuro)pharmaceuticals. Pharmaceutics. 2022;14(8):1729. 36015358 10.3390/pharmaceutics14081729 PMC9412499

[11] Bryniarski MA, Ren T, Rizvi AR, Snyder AM, Morris ME. Targeting the choroid plexuses for protein drug delivery. Pharmaceutics. 2020;12(10):963. 33066423 10.3390/pharmaceutics12100963 PMC7602164

[12] Liddelow SA. Development of the choroid plexus and blood–CSF barrier. Front Neurosci. 2015;9:32. 25784848 10.3389/fnins.2015.00032 PMC4347429

[13] Lauer AN, Tenenbaum T, Schroten H, Schwerk C. The diverse cellular responses of the choroid plexus during infection of the central nervous system. Am J Physiol Cell Physiol. 2018;314(2):C152–65. 29070490 10.1152/ajpcell.00137.2017

[14] Kaur C, Rathnasamy G, Ling EA. The choroid plexus in healthy and diseased brain. J Neuropathol Exp Neurol. 2016;75(3):198–213. 26888305 10.1093/jnen/nlv030

[15] Schwerk C, Tenenbaum T, Kim KS, Schroten H. The choroid plexus-a multi-role player during infectious diseases of the CNS. Front Cell Neurosci. 2015;9:80. 25814932 10.3389/fncel.2015.00080 PMC4357259

[16] Engelhardt B, Vajkoczy P, Weller RO. The movers and shapers in immune privilege of the CNS. Nat Immunol. 2017;18(2):123–31. 28092374 10.1038/ni.3666

[17] Meeker RB, Williams K, Killebrew DA, Hudson LC. Cell trafficking through the choroid plexus. Cell Adh Migr. 2012;6(5):390–6. 22902764 10.4161/cam.21054 PMC3496674

[18] Lopes Pinheiro MA, Kooij G, Mizee MR, Kamermans A, Enzmann G, Lyck R, et al. Immune cell trafficking across the barriers of the central nervous system in multiple sclerosis and stroke. Biochim Biophys Acta. 2016;1862(3):461–71. 26527183 10.1016/j.bbadis.2015.10.018

[19] Xu J, Ma C, Hua M, Li J, Xiang Z, Wu J. CNS and CNS diseases in relation to their immune system. Front Immunol. 2022;13:1063928. 36466889 10.3389/fimmu.2022.1063928 PMC9708890

[20] Solar P, Zamani A, Kubickova L, Dubovy P, Joukal M. Choroid plexus and the blood-cerebrospinal fluid barrier in disease. Fluids Barriers CNS. 2020;17(1):35. 32375819 10.1186/s12987-020-00196-2 PMC7201396

[21] Liu R, Zhang Z, Chen Y, Liao J, Wang Y, Liu J, et al. Choroid plexus epithelium and its role in neurological diseases. Front Mol Neurosci. 2022;15: 949231. 36340696 10.3389/fnmol.2022.949231 PMC9633854

[22] Wolburg H, Paulus W. Choroid plexus: biology and pathology. Acta Neuropathol. 2010;119(1):75–88. 20033190 10.1007/s00401-009-0627-8

[23] Thompson D, Brissette CA, Watt JA. The choroid plexus and its role in the pathogenesis of neurological infections. Fluids Barriers CNS. 2022;19(1):75. 36088417 10.1186/s12987-022-00372-6 PMC9463972

[24] Dando SJ, Mackay-Sim A, Norton R, Currie BJ, St John JA, Ekberg JA, et al. Pathogens penetrating the central nervous system: infection pathways and the cellular and molecular mechanisms of invasion. Clin Microbiol Rev. 2014;27(4):691–726. 25278572 10.1128/CMR.00118-13 PMC4187632

[25] Vandenhaute E, Stump-Guthier C, Lasierra Losada M, Tenenbaum T, Rudolph H, Ishikawa H, et al. The choroid plexus may be an underestimated site of tumor invasion to the brain: an in vitro study using neuroblastoma cell lines. Cancer Cell Int. 2015;15:102. 26500454 10.1186/s12935-015-0257-2 PMC4619509

[26] Erb U, Schwerk C, Schroten H, Karremann M. Review of functional in vitro models of the blood-cerebrospinal fluid barrier in leukaemia research. J Neurosci Methods. 2020;329: 108478. 31669338 10.1016/j.jneumeth.2019.108478

[27] Demeestere D, Libert C, Vandenbroucke RE. Therapeutic implications of the choroid plexus-cerebrospinal fluid interface in neuropsychiatric disorders. Brain Behav Immun. 2015;50:1–13. 26116435 10.1016/j.bbi.2015.06.010

[28] Gath U, Hakvoort A, Wegener J, Decker S, Galla HJ. Porcine choroid plexus cells in culture: expression of polarized phenotype, maintenance of barrier properties and apical secretion of CSF-components. Eur J Cell Biol. 1997;74(1):68–78. 9309392

[29] Haselbach M, Wegener J, Decker S, Engelbertz C, Galla HJ. Porcine Choroid plexus epithelial cells in culture: regulation of barrier properties and transport processes. Microsc Res Tech. 2001;52(1):137–52. 11135456 10.1002/1097-0029(20010101)52:1<137::AID-JEMT15>3.0.CO;2-J

[30] Strazielle N, Ghersi-Egea J-F. In vitro models of the blood-cerebrospinal fluid barrier and their use in neurotoxicological research. NeuroMethods. 2011;56:161–84. 10.1007/978-1-61779-077-5_8

[31] Lallai V, Ahmed A, Fowler CD. Method for primary epithelial cell culture from the rat choroid plexus. Bio Protoc. 2020;10(4): e3532. 33654756 10.21769/BioProtoc.3532 PMC7842687

[32] Delery EC, MacLean AG. Culture model for non-human primate choroid plexus. Front Cell Neurosci. 2019;13:396. 31555096 10.3389/fncel.2019.00396 PMC6724611

[33] Kitazawa T, Hosoya K, Watanabe M, Takashima T, Ohtsuki S, Takanaga H, et al. Characterization of the amino acid transport of new immortalized choroid plexus epithelial cell lines: a novel in vitro system for investigating transport functions at the blood-cerebrospinal fluid barrier. Pharm Res. 2001;18(1):16–22. 11336348 10.1023/A:1011014424212

[34] Shi LZ, Zheng W. Establishment of an in vitro brain barrier epithelial transport system for pharmacological and toxicological study. Brain Res. 2005;1057(1–2):37–48. 16126179 10.1016/j.brainres.2005.07.046 PMC4151265

[35] Zheng W, Zhao Q. Establishment and characterization of an immortalized Z310 choroidal epithelial cell line from murine choroid plexus. Brain Res. 2002;958(2):371–80. 12470873 10.1016/S0006-8993(02)03683-1 PMC3980880

[36] Kumabe T, Tominaga T, Kondo T, Yoshimoto T, Kayama T. Intraoperative radiation therapy and chemotherapy for huge choroid plexus carcinoma in an infant–case report. Neurol Med Chir (Tokyo). 1996;36(3):179–84. 8869156 10.2176/nmc.36.179

[37] Ishiwata I, Ishiwata C, Ishiwata E, Sato Y, Kiguchi K, Tachibana T, et al. Establishment and characterization of a human malignant choroids plexus papilloma cell line (HIBCPP). Hum Cell. 2005;18(1):67–72. 16130902 10.1111/j.1749-0774.2005.tb00059.x

[38] Schroten M, Hanisch FG, Quednau N, Stump C, Riebe R, Lenk M, et al. A novel porcine in vitro model of the blood-cerebrospinal fluid barrier with strong barrier function. PLoS ONE. 2012;7(6): e39835. 22745832 10.1371/journal.pone.0039835 PMC3382175

[39] Schwerk C, Papandreou T, Schuhmann D, Nickol L, Borkowski J, Steinmann U, et al. Polar invasion and translocation of neisseria meningitidis and *Streptococcus* suis in a novel human model of the blood-cerebrospinal fluid barrier. PLoS ONE. 2012;7(1): e30069. 22253884 10.1371/journal.pone.0030069 PMC3256222

[40] Tenenbaum T, Papandreou T, Gellrich D, Friedrichs U, Seibt A, Adam R, et al. Polar bacterial invasion and translocation of *Streptococcus* suis across the blood-cerebrospinal fluid barrier in vitro. Cell Microbiol. 2009;11(2):323–36. 19046337 10.1111/j.1462-5822.2008.01255.x

[41] Dinner S, Borkowski J, Stump-Guthier C, Ishikawa H, Tenenbaum T, Schroten H, et al. A choroid plexus epithelial cell-based model of the human blood-cerebrospinal fluid barrier to study bacterial infection from the basolateral side. J Vis Exp: JoVE. 2016. 10.3791/54061. 27213495 10.3791/54061 PMC4942071

[42] Augustin HG, Koh GY. Organotypic vasculature: from descriptive heterogeneity to functional pathophysiology. Science (New York, NY). 2017. 10.1126/science.aal2379. 10.1126/science.aal2379 28775214

[43] Denzer L, Muranyi W, Schroten H, Schwerk C. The role of PLVAP in endothelial cells. Cell Tissue Res. 2023;392(2):393–412. 36781482 10.1007/s00441-023-03741-1 PMC10172233

[44] Pacitti D, Privolizzi R, Bax BE. Organs to cells and cells to organoids: the evolution of in vitro central nervous system modelling. Front Cell Neurosci. 2019;13:129. 31024259 10.3389/fncel.2019.00129 PMC6465581

[45] Pritchard JB, Sweet DH, Miller DS, Walden R. Mechanism of organic anion transport across the apical membrane of choroid plexus. J Biolo Chem. 1999;274(47):33382–7. 10.1074/jbc.274.47.33382 10559217

[46] Wang X, Miller DS, Zheng W. Intracellular localization and subsequent redistribution of metal transporters in a rat choroid plexus model following exposure to manganese or iron. Toxicol Appl Pharmacol. 2008;230(2):167–74. 18420243 10.1016/j.taap.2008.02.024 PMC2586425

[47] Villalobos AR, Miller DS, Renfro JL. Transepithelial organic anion transport by shark choroid plexus. Am J Physiol Regul Integr Comp Physiol. 2002;282(5):R1308–16. 11959670 10.1152/ajpregu.00677.2001

[48] Breen CM, Sykes DB, Fricker G, Miller DS. Confocal imaging of organic anion transport in intact rat choroid plexus. Am J Physiol Renal Physiol. 2002;282(5):F877–85. 11934698 10.1152/ajprenal.00171.2001

[49] Ulloa V, Saldivia N, Ferrada L, Salazar K, Martinez F, Silva-Alvarez C, et al. Basal sodium-dependent vitamin C transporter 2 polarization in choroid plexus explant cells in normal or scorbutic conditions. Sci Rep. 2019;9(1):14422. 31594969 10.1038/s41598-019-50772-2 PMC6783570

[50] Petersen N, Torz L, Jensen KHR, Hjorto GM, Spiess K, Rosenkilde MM. Three-dimensional explant platform for studies on choroid plexus epithelium. Front Cell Neurosci. 2020;14:108. 32431599 10.3389/fncel.2020.00108 PMC7214744

[51] Watanabe M, Kang YJ, Davies LM, Meghpara S, Lau K, Chung CY, et al. BMP4 sufficiency to induce choroid plexus epithelial fate from embryonic stem cell-derived neuroepithelial progenitors. J Neurosci. 2012;32(45):15934–45. 23136431 10.1523/JNEUROSCI.3227-12.2012 PMC3505486

[52] Sakaguchi H, Kadoshima T, Soen M, Narii N, Ishida Y, Ohgushi M, et al. Generation of functional hippocampal neurons from self-organizing human embryonic stem cell-derived dorsomedial telencephalic tissue. Nat Commun. 2015;6:8896. 26573335 10.1038/ncomms9896 PMC4660208

[53] Huch M, Knoblich JA, Lutolf MP, Martinez-Arias A. The hope and the hype of organoid research. Development. 2017;144(6):938–41. 28292837 10.1242/dev.150201

[54] Lancaster MA, Renner M, Martin CA, Wenzel D, Bicknell LS, Hurles ME, et al. Cerebral organoids model human brain development and microcephaly. Nature. 2013;501(7467):373–9. 23995685 10.1038/nature12517 PMC3817409

[55] Lancaster MA, Knoblich JA. Generation of cerebral organoids from human pluripotent stem cells. Nat Protoc. 2014;9(10):2329–40. 25188634 10.1038/nprot.2014.158 PMC4160653

[56] Pellegrini L, Bonfio C, Chadwick J, Begum F, Skehel M, Lancaster MA. Human CNS barrier-forming organoids with cerebrospinal fluid production. Science (New York, NY). 2020. 10.1126/science.aaz5626. 10.1126/science.aaz5626 PMC7116154 32527923

[57] Shaker MR, Slonchak A, Al-Mhanawi B, Morrison SD, Sng JDJ, Cooper-White J, et al. Choroid plexus defects in Down syndrome brain organoids enhance neurotropism of SARS-CoV-2. Sci Adv. 2024;10(23):eadj4735. 38838150 10.1126/sciadv.adj4735 PMC11152128

[58] Ye B. Approaches to vascularizing human brain organoids. PLoS Biol. 2023;21(5): e3002141. 37155714 10.1371/journal.pbio.3002141 PMC10194923

[59] Aazmi A, Zhou H, Lv W, Yu M, Xu X, Yang H, et al. Vascularizing the brain in vitro. iScience. 2022;25(4): 104110. 35378862 10.1016/j.isci.2022.104110 PMC8976127

[60] Ingber DE. Human organs-on-chips for disease modelling, drug development and personalized medicine. Nat Rev Genet. 2022;23(8):467–91. 35338360 10.1038/s41576-022-00466-9 PMC8951665

[61] Zhou Y, Qiao H, Xu F, Zhao W, Wang J, Gu L, et al. Bioengineering of a human physiologically relevant microfluidic blood-cerebrospinal fluid barrier model. Lab Chip. 2023;23(13):3002–15. 37291941 10.1039/D3LC00131H

[62] Lim J, Rhee S, Choi H, Lee J, Kuttappan S, Yves Nguyen TT, et al. Engineering choroid plexus-on-a-chip with oscillatory flow for modeling brain metastasis. Mater Today Bio. 2023;22: 100773. 37664794 10.1016/j.mtbio.2023.100773 PMC10474164

[63] Tan SY, Feng X, Cheng LKW, Wu AR. Vascularized human brain organoid on-chip. Lab Chip. 2023;23(12):2693–709. 37256563 10.1039/D2LC01109C

[64] Xu H, Lehtinen MK. Choroid plexus organoids: harnessing CSF gatekeepers for brain therapeutics. Cell Stem Cell. 2020;27(2):191–2. 32763178 10.1016/j.stem.2020.07.009

[65] Pellegrini L, Lancaster MA. Breaking the barrier: in vitro models to study choroid plexus development. Curr Opin Cell Biol. 2021;73:41–9. 34182208 10.1016/j.ceb.2021.05.005

[66] Dahm T, Rudolph H, Schwerk C, Schroten H, Tenenbaum T. Neuroinvasion and inflammation in viral central nervous system infections. Mediat Inflamm. 2016;2016:8562805. 10.1155/2016/8562805 PMC4897715 27313404

[67] Kim J, Alejandro B, Hetman M, Hattab EM, Joiner J, Schroten H, et al. Zika virus infects pericytes in the choroid plexus and enters the central nervous system through the blood-cerebrospinal fluid barrier. PLoS Pathog. 2020;16(5): e1008204. 32357162 10.1371/journal.ppat.1008204 PMC7194358

[68] Su X, Yue P, Kong J, Xu X, Zhang Y, Cao W, et al. Human brain organoids as an in vitro model system of viral infectious diseases. Front Immunol. 2021;12: 792316. 35087520 10.3389/fimmu.2021.792316 PMC8786735

[69] Speidel A, Theile M, Pfeiffer L, Herrmann A, Figarella K, Ishikawa H, et al. Transmigration of Trypanosoma brucei across an in vitro blood-cerebrospinal fluid barrier. iScience. 2022;25(4):104014. 35313698 10.1016/j.isci.2022.104014 PMC8933718

[70] Jacob F, Pather SR, Huang WK, Zhang F, Wong SZH, Zhou H, et al. Human pluripotent stem cell-derived neural cells and brain organoids reveal SARS-CoV-2 neurotropism predominates in choroid plexus epithelium. Cell Stem Cell. 2020;27(6):937-50 e9. 33010822 10.1016/j.stem.2020.09.016 PMC7505550

[71] Pellegrini L, Albecka A, Mallery DL, Kellner MJ, Paul D, Carter AP, et al. SARS-CoV-2 infects the brain choroid plexus and disrupts the blood-CSF barrier in human brain organoids. Cell Stem Cell. 2020;27(6):951-61 e5. 33113348 10.1016/j.stem.2020.10.001 PMC7553118

[72] Tenenbaum T, Steinmann U, Friedrich C, Berger J, Schwerk C, Schroten H. Culture models to study leukocyte trafficking across the choroid plexus. Fluids Barriers CNS. 2013;10(1):1. 23305147 10.1186/2045-8118-10-1 PMC3560101

[73] Bernd A, Ott M, Ishikawa H, Schroten H, Schwerk C, Fricker G. Characterization of efflux transport proteins of the human choroid plexus papilloma cell line HIBCPP, a functional in vitro model of the blood-cerebrospinal fluid barrier. Pharm Res. 2015;32(9):2973–82. 25986174 10.1007/s11095-015-1679-1

[74] Hulme L, Hochstetler A, Schwerk C, Schroten H, Ishikawa H, Tung CY, et al. Characterization of TRPV4-mediated signaling pathways in an optimized human choroid plexus epithelial cell line. Am J Physiol Cell Physiol. 2022;323(6):C1823–42. 35938676 10.1152/ajpcell.00193.2022 PMC9744646

[75] Muok L, Liu C, Chen X, Esmonde C, Arthur P, Wang X, et al. Inflammatory response and exosome biogenesis of choroid plexus organoids derived from human pluripotent stem cells. Int J Mol Sci. 2023;24(8):7660. 37108817 10.3390/ijms24087660 PMC10146825

[76] Lyu Z, Park J, Kim KM, Jin HJ, Wu H, Rajadas J, et al. A neurovascular-unit-on-a-chip for the evaluation of the restorative potential of stem cell therapies for ischaemic stroke. Nat Biomed Eng. 2021;5(8):847–63. 34385693 10.1038/s41551-021-00744-7 PMC8524779

[77] Alcendor DJ, Block FE 3rd, Cliffel DE, Daniels JS, Ellacott KL, Goodwin CR, et al. Neurovascular unit on a chip: implications for translational applications. Stem Cell Res Ther. 2013. 10.1186/scrt379. 24564885 10.1186/scrt379 PMC4029462

[78] Smirnova L, Hartung T. The promise and potential of brain organoids. Adv Healthc Mater. 2024. 10.1002/adhm.202302745. 38252094 10.1002/adhm.202302745

